# Preparation of a Gradient Anti-Oxidation Coating for Aircraft C/C Composite Brake Disc and Its High-Temperature In Situ Self-Healing Performance

**DOI:** 10.3390/ma17102344

**Published:** 2024-05-15

**Authors:** Dan Zheng, Haiqing Yin

**Affiliations:** 1 Institute for Advanced Materials and Technology, University of Science and Technology Beijing, Beijing 100083, China; 2 Beijing Bei Mo Gao Ke Friction Material Co., Ltd., Beijing 102206, China; 3 Collaborative Innovation Center of Steel Technology, University of Science and Technology Beijing, Beijing 100083, China

**Keywords:** aircraft brake disc, C/C composite, anti-oxidation coating, self-healing, high-temperature in situ

## Abstract

We studied a gradient anti-oxidation coating of C/C composite materials for aircraft brake discs with a simple process and low costs. The gradient coating consists of two layers, of which the inner layer is prepared with tetraethyl orthosilicate (Si (OC2H5)4), C_2_H_5_OH, H_3_PO_4_ and B_4_C, and the outer layer is prepared with Na_2_B_4_O_7_.10H_2_O, B_2_O_3_, and SiO_2_ powder. The experimental results show that after being oxidized at 700 °C for 15 h, the oxidation weight loss of the sample with the coating was only −0.17%. At the same time, after 50 thermal cycles in air at 900 °C, the sample’s oxidation weight loss was only −0.06%. We conducted the 1:1 dynamic simulation test for aircraft brake discs, and the brake disc did not oxidize, thus meeting the requirements for aircraft use. In addition, the anti-oxidation mechanism of the coating was analyzed via scanning electron microscopy (SEM), X-ray diffraction (XRD), differential thermal analysis (DSC-TGA), and high-temperature in situ SEM.

## 1. Introduction

C/C materials are carbon-fiber-reinforced carbon matrix composites, with a high specific modulus and specific strength. They have good thermal physical properties and friction and wear properties, so they can meet the requirements for aircraft brakes well: lightweight, long life, smooth braking, fast heat dissipation, high specific heat capacity, small thermal expansion, good thermal shock resistance, and other requirements [1,2]. These advantages lead it to be widely used in aircraft wheel brake materials. In an inert atmosphere, C/C composite materials can remain stable at up to 2000 °C. However, without protective measures, they will begin to oxidize at 400 °C in air, which can affect the brake disc’s efficiency and even lead to failure [3,4,5]. The use temperature for C/C composite materials for aircraft brake discs is generally 700–800 °C, which can reach 1000 °C when interrupting takeoff. In addition, brake discs need to be used frequently, and C/C materials must have good thermal shock resistance [6,7].

Aircraft brake discs mainly use phosphate or B_2_O_3_ as an anti-oxidation coating. However, the coefficient of thermal expansion (CTE) of these coatings is much higher than that of C/C matrixes, which can result in many microcracks in the coating [8,9,10]. Phosphate-based coatings cannot effectively heal these cracks, and although B_2_O_3_ has a good self-healing ability, its oxidation products are easy to volatilize at high temperatures, which affects its anti-oxidation performance [11,12,13]. At present, the anti-oxidation research on C/C composites is more in the anti-ablation or material modification of structural materials and is not applicable to aircraft brake materials [14,15,16,17,18,19]. There are few public literature reports on the in situ high-temperature self-healing performance of coatings or verifications of anti-oxidation performance through brake disc product braking tests according to CTSO-135a [20,21,22]. This article focuses on the requirements for aircraft brake disc usage and studies a new type of boron silicon anti-oxidation gradient coating with a simple process, a low cost, and good anti-oxidation performance. It can achieve self-healing at high temperatures and meet the requirements for aircraft use.

## 2. Materials and Methods

### 2.1. Materials and Reagents

The raw materials required for the experiment and their main technical indicators are shown in Table 1.

**Figure 1:**
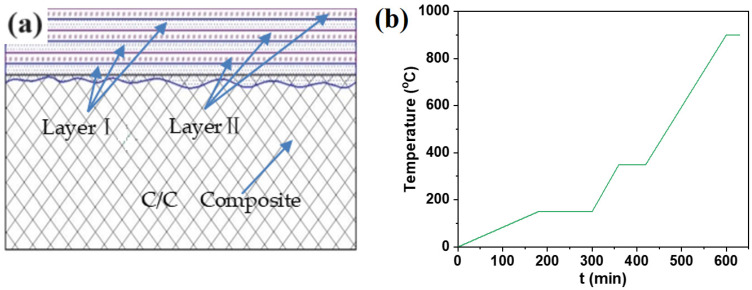
(**a**) Schematic diagram of alternating coating; (**b**) pretreatment temperature curve.

Figure 1a shows a gradient coating brushing schematic and the pretreatment temperature curve. Preparing the coating involves two steps: application and pretreatment. Step 1: we brushed the Layer I coating solution containing tetraethyl orthosilicate, B_4_C, and other components in Table 1 evenly on the surface of the sample, and then brushed the Layer II coating solution containing SiO_2_, B_2_O_3_, and other components in Table 1. We alternately painted three times. Step 2: the coated sample was pretreated under N_2_ protection, according to the temperature curve in Figure 1b.

### 2.2. Specimen Preparation

The C/C composite material used for aircraft brake discs is made through a chemical vapor deposition (CVD) process, with a density of 1.78 g/cm^3^. The static and dynamic anti-oxidation performance sample in this study was directly cut from the aircraft brake disc, with a specification of 10 × 10 × 10 mm^3^. It was cleaned with alcohol and dried at 120 °C for 2 h, then the gradient coating and pretreatment was applied as described in Section 2.2. High-temperature in situ SEM and XRD were performed on the gradient-coated samples after static oxidation. DSC-TGA samples were coated with a gradient coating mixture; that is, the coating solution was put in a beaker in proportion and mixed well. After natural drying, the dry solids in the beaker were placed in a mortar to obtain the coating mixture powder. By directly using aircraft brake disc products consisting of 4 rotor discs, 3 static discs, 1 bearing disc, and 1 compression disc, we were able to conduct a 1:1 dynamic brake simulation test together with the brake device, wheels, and tires. After cleaning, these samples were dried for 2 h at 120 °C, and the non-friction surface was coated with gradient coating and pretreated under N_2_ protection.

### 2.3. Oxidation Tests

We tested the coating with a constant temperature static oxidation test in a Muffle furnace; the testing temperatures were 700 °C, 900 °C, and 1000 °C. A dynamic oxidation test of the coating was completed in the Muffle furnace according to the following process: when the temperature reached 900 °C, the sample was placed in the furnace for 5 min, transferred to a room temperature environment for 5 min, and then placed back in the furnace. We repeated this cycle 50 times. We weighed the sample at room temperature using a BS100S electronic balance: maximum load, 100 g; sensitivity, 0.1 mg. The percentage of oxidation weight loss was calculated according to the following formula. W_0_ is the pre-oxidation mass; W_1_ is the post-oxidation mass.

(1) $$\delta \%=\frac{W_{0}-W_{1}}{W_{0}}\times 100\%$$

### 2.4. Characterization

**Figure 2:**
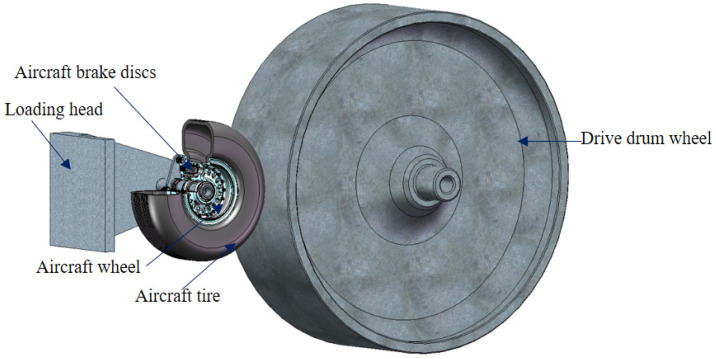
Working principle of BMJ-1 dynamic simulation test bench.

The gradient coating powder with 90 μL was analyzed via DSC-TGA under an air atmosphere with an SDT~Q600 thermogravimetric analyzer (TA Instruments, New Castle, DE, USA). The heating rate was 10 °C/min, and the temperature range was 30–1000 °C. A Rikkyo TTR3 X-ray diffractometer (Tokyo, Japan) was used to analyze the phase of the coating before and after oxidation. Its maximum rated output power is 18 KW, the tube voltage is 20–60 kV, the tube current is 10–300 mA, the anode is Cu, the scanning speed is 4°/min, and the scanning range is 10°–90°. A LEO 1450 scanning electron microscope (Zeiss, Oberkochen, Germany) was used to analyze the morphology of the coating surface before and after oxidation. The surface morphology and self-healing properties of the coating at different temperatures were analyzed with a TESCAN S8000 (Tescan, Brno, Czech Republic) in situ high-temperature scanning electron microscope. Figure 2 shows the BMJ-01 aircraft wheels and tires 1:1 dynamic simulation test bench, which can fully simulate the actual braking conditions of the aircraft, and the brake test is completed according to CTSO-135a to verify the oxidation resistance of the aircraft brake discs.

## 3. Results and Discussion

### 3.1. Microstructures of the Coating

**Figure 3:**
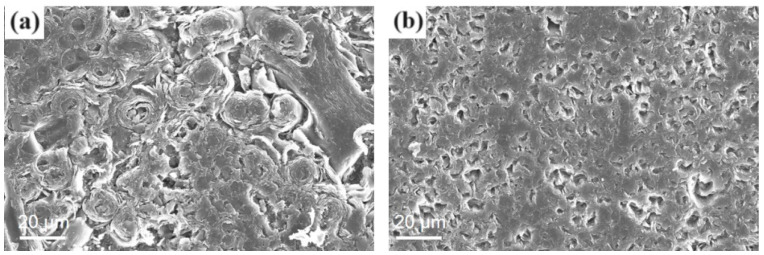
Microscopic morphology of C/C composite materials: (**a**) cross-section; (**b**) vertical section.

Figure 3 shows the transverse and longitudinal morphology of the C/C composite matrix. A C/C composite material is composed of carbon fiber and deposited carbon, and there are pores on its surface and inside its matrix.

Therefore, the coating solution was able to penetrate a certain depth of the pores when brushed, and it integrated with the matrix after pretreatment so that the combination would be firmer.

**Figure 4:**
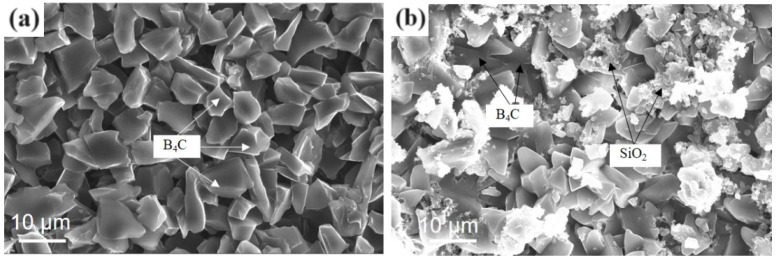
Micromorphology after coating with layer solution: (**a**) Layer I; (**b**) Layer II.

Figure 4a shows the microscopic morphology after Layer I solution coating. The tetraethyl orthosilicate in the Layer I solution was hydrolyzed with ethanol as a solvent and phosphoric acid as a catalyst.

The hydrolysis of tetraethyl orthosilicate is a widely used sol–gel technique for preparing new materials such as glass and ceramics with SiO_2_ as the matrix material [23,24].

The hydrolysis reaction of tetraethyl orthosilicate is shown in Equation (2):
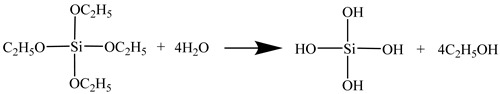
(2)

Through hydrolysis, tetraethyl orthosilicate forms sol through polycondensation, forming a wet gel with a polycondensation reaction, as per Equation (3):
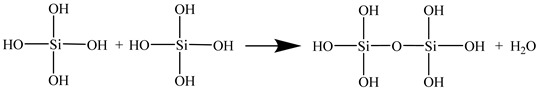
(3)

The dehydration reaction of the coating during the high-temperature pretreatment is shown in Formula (4):
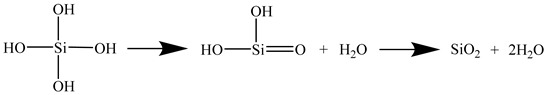
(4)

Tetraethyl orthosilicate hydrolysate was mixed with B_4_C powder in a beaker and continuously stirred; at this time, the solution state was a black suspension. We applied the Layer I solution to all outer surfaces of the 10 × 10 × 10 mm^3^ sample block. The solution penetrated the matrix through the porous part of the material; the rest of the solution containing B_4_C powder was coated on the surface of the matrix, and the B_4_C powder filled the pores of the material and formed a Layer I solution coating on the surface of the matrix after natural drying.

Figure 4b shows the microscopic morphology after the Layer II solution was coated on the surface of the Layer I solution. The Layer II solution was composed of Na_2_B_4_O_7_.10H_2_O, B_2_O_3_, SiO_2_ powder, and deionized water, and it was dissolved through stirring it in a water bath at 60 °C. B_2_O_3_ dissolves in water to form boric acid, and its solubility in water increases with increasing temperatures. Na_2_B_4_O_7_.10H_2_O is a colorless translucent crystal or white crystalline powder, soluble in water and weakly alkaline in an aqueous solution. It was mixed with SiO_2_ powder to form a micro-white suspension.

**Figure 5:**
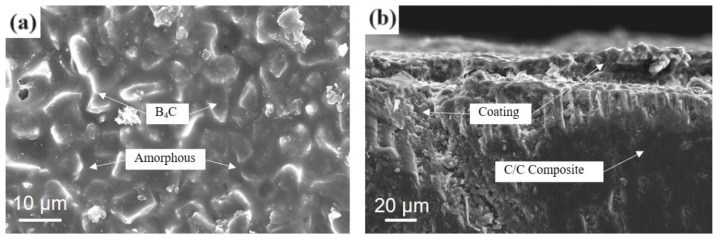
Micromorphology of gradient coating pretreatment: (**a**) outer surface; (**b**) cross-section.

Figure 5a shows the microstructure of the gradient coating formed after the Layer I and Layer II solutions were alternatingly brushed three times and pretreated according to the temperature curve in Figure 1b.

The gradient coating formed a continuous molten coating layer after pretreatment, which was wrapped in B_4_C particles. Figure 5b is a sectional SEM diagram of the gradient coating sample. The coating solution penetrated the matrix pores of the C/C composite material during brushing, and after pretreatment, firmly bonded with the matrix to form a whole. The SiO_2_ generated after hydrolyzing tetraethyl orthosilicate infiltrated the matrix pores, which improved the thermal expansion adaptability of the coating and the matrix, reducing the interface stress; consequently, this increased the bonding strength of the coating. At the same time, the wettability of the interface between the coating and the substrate was improved, which is conducive to the coating brushing operation. In addition to filling the pores of the material, B_4_C particles can react with oxygen to form B_2_O_3_ under high-temperature conditions, which plays a role in protecting the material matrix and healing microcracks.

### 3.2. Oxidation Behaviors of the Coating

**Figure 6:**
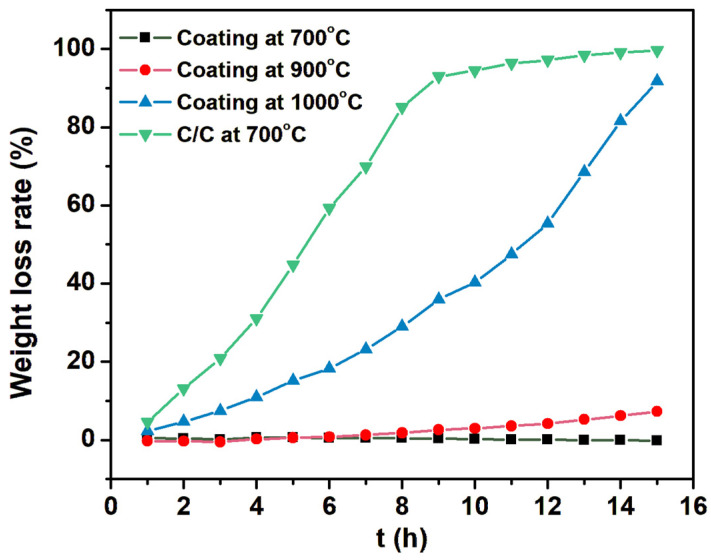
Oxidation weight loss time curves for C/C samples at 700 °C and gradient-coated samples at 700 °C, 900 °C, and 1000 °C for 15 h.

Figure 6 shows the oxidation weight loss rate–time curve of the uncoated C/C composite material sample and the gradient coating sample over 15 h of static oxidation at different temperatures.

The weight loss of the uncoated C/C composite material samples increased sharply by more than 70% after 7 h of oxidation in static air at 700 °C, and the weight loss rate approached 99% after 15 h of oxidation. The C/C composite material with the gradient coating that was oxidized in static air at 700 °C for 15 h had a weight loss rate of −0.17%; this negative weight loss rate is due to weight gain after B_4_C oxidizes into B_2_O_3_. At present, the oxidative weight loss rates of phosphate coating and composite coating widely used in C/C composite brake discs are about 4% and 1%, respectively, at 700 °C for 10 h. The test results show that the gradient coating has a continuous antioxidant effect at 700 °C [19,25,26]. The weight loss rate of the gradient coating was 7.23% in static air at 900 °C for 15 h. During the 4 h of 1000 °C static air oxidation, the weight loss rate was still less than 10%; it continued to oxidize, and the weight loss rate continued to rise.

To evaluate the comprehensive performance of the gradient coating, it is also necessary to consider the actual working conditions, such as cooling after completing the aircraft C/C composite brake disc and aircraft braking. Therefore, the dynamic oxidation resistance of the coating at high temperatures was also tested. The 12 samples with gradient coating were oxidized for 5 min in a static air environment of 900 °C and cooled for 5 min at room temperature after taking out the sample for one cycle, and fifty cycles were carried out continuously. The test results for the dynamic antioxidant properties are shown in Table 2. After 50 consecutive cycles of 900 °C~room temperature, the coating and the substrate did not flake off, and basically, no weight loss occurred. Compared with the thermal shock weight loss rate of more than 2% for phosphate coating and 1% for composite coating of existing brake discs [19,25,26], the gradient coating has a better thermal shock resistance.

### 3.3. Microstructures of the Coating after Oxidation

**Figure 7:**
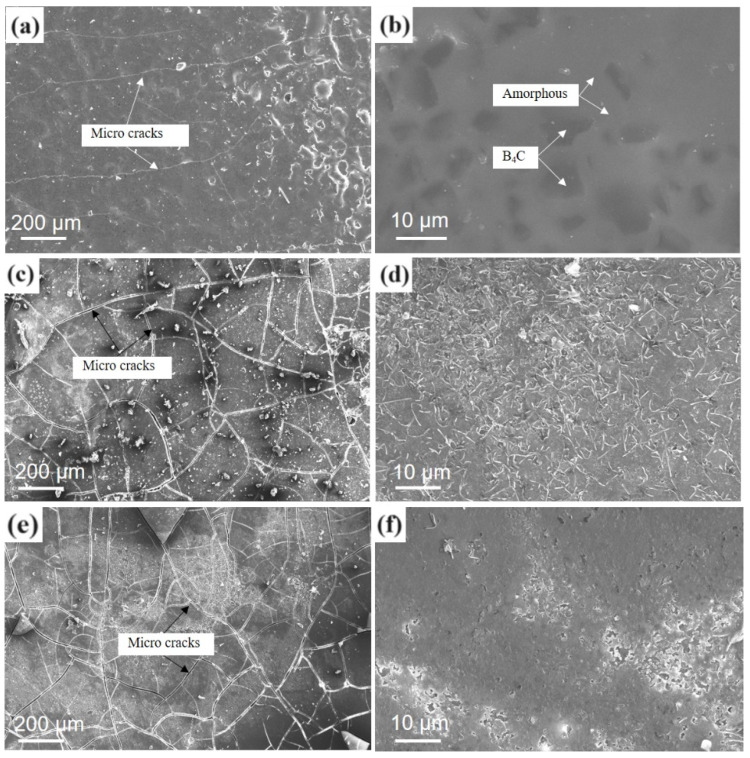
Surface microstructure of the coating after oxidation and cooling: (**a**,**b**) at 700 °C; (**c**,**d**) at 900 °C; (**e**,**f**) at 1000 °C.

Figure 7 shows the microstructures of the gradient coating after oxidation for 2 h and cooling in static air at 700 °C (Figure 7a,b), 900 °C (Figure 7c,d), and 1000 °C (Figure 7e,f).

After oxidation at 700 °C, the gradient coating formed a relatively continuous amorphous phase and covered the B_4_C particles, but there were fine cracks in different directions on the surface of the tissue. These microcracks were caused by a difference in CTE between the fluid amorphous phase formed at high temperatures and the C/C composite matrix after cooling [27,28,29]. After oxidation at 900 °C and 1000 °C, B_4_C underwent an oxidation reaction, and its particles were no longer obvious; however, the amorphous phase that formed was denser, and the cracks after cooling were more obvious. If the coating is oxidized again at a high temperature, an amorphous phase with fluidity will form again, such that the crack will heal and prevent oxygen from entering the matrix pores to oxidize the material.

### 3.4. The Anti-Oxidation Mechanism of the Coating

**Figure 8:**
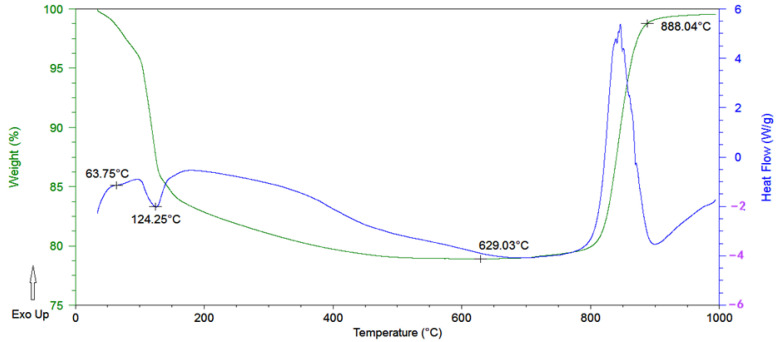
DSC-TGA curve of gradient coating powder in air.

Figure 8 shows the DSC-TGA curve of the gradient coating powder when oxidized in static air. The curve shows that the oxidation weight loss of the coating can be divided into three stages.

The first stage is the dehydration stage: The coating begins to lose weight at 50 °C mainly because of the physical evaporation of solvent water. At about 63 °C, there is a slow endothermic slope, at which point Na_2_B_4_O_7_.10H_2_O is dehydrated to Na_2_B_4_O_7_.5 H_2_O. At 70–100 °C, the solvent water continues to evaporate. At about 124 °C, there is an obvious endothermic peak; at this time, Na_2_B_4_O_7_.5 H_2_O is dehydrated to Na_2_B_4_O_7_.2 H_2_O. Heated to about 350 °C, H_3_BO_3_ decomposes into B_2_O_3_ and H_2_O. When heating is continued to 400–500 °C, Na_2_B_4_O_7_.2 H_2_O can be dehydrated into Na_2_B_4_O_7_ [30,31,32].

The second stage is the stable stage: When the temperature rises to around 600 °C, B_2_O_3_ in the second-layer coating solution begins to evaporate after melting, and the B_4_C oxidation reaction has not yet fully begun. Therefore, the weight loss rate of the coating reaches its highest point. When the temperature continues to rise to 800 °C, the coating experiences almost no weight loss and in fact gains a little weight, indicating that the dehydration transformation is complete; B_4_C begins to undergo an oxidation reaction, resulting in glassy B_2_O_3_ and weight gain [33,34,35].

(5) $$B_{4}C+4O_{2}\to 2B_{2}O_{3}+CO_{2}$$

The third stage is the weight gain period: The exothermic peak appearing at 800–900 °C shows that B_4_C is completely oxidized into forming glassy B_2_O_3_ and the weight is greatly increased. After 900 °C, the coating no longer gains weight and remains in a molten state. At this time, SiO_2_ and B_2_O_3_ dissolve into a B_2_O_3_-SiO_2_ glass phase, which has suitable fluidity at 600 °C to 1000 °C. It can not only improve the viscosity of the coating, avoiding high-temperature volatilization and brake rotation, it can also seal cracks and pores on the surface of the material.

**Figure 9:**
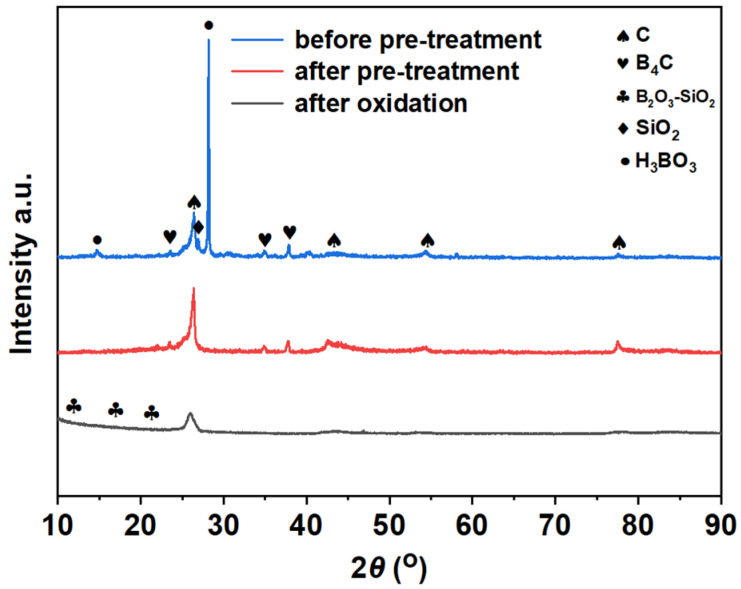
X-ray diffraction analysis of the coating.

Figure 9 shows the X-ray diffraction analysis of gradient coatings before pre-treatment, after pre-treatment, and after oxidation at 1000 °C. Before pretreatment, the diffraction peaks of C/C matrix, B_4_C and SiO_2_, as well as diffraction peaks of H_3_BO_3_ formed by B_2_O_3_ dissolved in water, can be observed. After pre-treatment: Due to N_2_ protection during the pre-treatment process, B_4_C does not undergo oxidation, so its diffraction peak can still be observed. H_3_BO_3_ decomposes into B_2_O_3_ and H_2_O at high temperatures, with a portion of B_2_O_3_ melting to form boron oxide glass and a portion forming B_2_O_3_-SiO_2_ glass phase with SiO_2_. After oxidation at 1000 °C, the diffraction peak of B_4_C disappears due to complete oxidation to B_2_O_3_, and a B_2_O_3_-SiO_2_ glass phase is formed [36,37,38,39].

**Figure 10:**
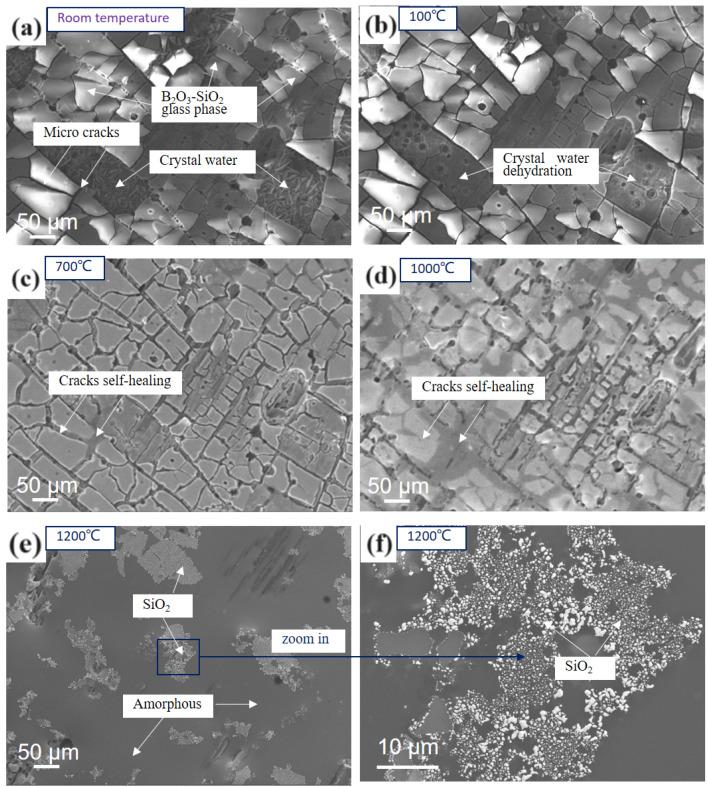
In situ SEM microstructure of the oxidized coating heated from room temperature to 1200 °C: (**a**) at room temperature; (**b**) at 100 °C; (**c**) at 700 °C (**d**) at 1000 °C (**e**) at 1200 °C; (**f**) zoom in at 1200 °C.

Figure 10 shows the in situ SEM microstructure of the gradient coating oxidized in air at a high temperature, from room temperature to 1200 °C under a N_2_ atmosphere.

Figure 10 shows the in situ SEM microstructure of an oxidized gradient coating heated from room temperature to 1200 °C in an N_2_ atmosphere. Figure 10a shows the surface of the oxidized gradient coating is largely B_2_O_3_-SiO_2_ glass at room temperature with micro cracks and partial spalling between the blocks, and also shows the fibrous crystal water adsorbed on the bottom layer after spalling. After heating up to 100 °C, the crystal water decomposes and dehydrates (Figure 10b). When the temperature rises to 700 °C, the flow of molten boron glass begins to appear in microcracks between the blocks (Figure 10c). When the temperature rises to 1000 °C, the fluidity of the boron glass increases, and part of the borosilicate glass begins to melt, jointly filling the pores and blocking oxygen from eroding the matrix (Figure 10d). Heating to 1200 °C results in the complete melting of the coating’s surface, resulting in a lower viscosity and greater fluidity (Figure 10e). Figure 10f shows that after the borosilicate glass is completely melted at 1200 °C, the borosilicate glass forms one body, and the white SiO_2_ particles form agglomerations.

### 3.5. 1:1 Dynamic Simulation Test of Aircraft Brake Discs

**Figure 11:**
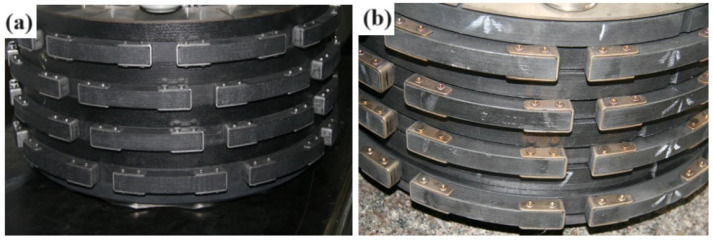
Appearance of aircraft brake discs before and after a 1:1 dynamic simulation test: (**a**) before the test; (**b**) after the test.

Figure 11 shows C/C composite brake discs coated with gradient coating before and after a 1:1 dynamic simulation test. Before the entire test, the brake discs were kept at −55 °C for 72 h then returned to room temperature. After 108 instances of landing braking (braking temperature range: 700–750 °C), one dry condition Reject Take Off brake test (1057 °C), one dry condition Reject Take Off brake test with extreme thin brake discs (1074 °C), and one wet condition Reject Take Off brake test (1127.6 °C) are performed. After these brake tests, the brake disc had a good appearance and did not incur oxidation or damage.

## 4. Conclusions

1. An anti-oxidation gradient coating for aircraft C/C composite brake discs was prepared. The coating is uniform and dense, has a high bonding strength, and has good anti-oxidation and thermal shock resistance. After oxidation at 700 °C for 15 h in air, the oxidation weight loss rate of the sample was −0.17%. After 50 instances of thermal cycling in air at 900 °C, the oxidation weight loss rate of the sample was −0.06%.
2. The corresponding oxidation curve of the coating in the oxidation process can be divided into three stages, namely, the coating dehydration period, the stable period, and the weight gain period. In the oxidation process, tetraethyl orthosilicate in the coating can be hydrolyzed into SiO_2_ and form a good combination with the substrate. B_4_C can oxidize to form glassy B_2_O_3_, which, together with SiO_2_ in the coating component, forms a dense borosilicate glass. It can effectively fill cracks and pores, thereby preventing the invasion of oxygen and reducing the volatilization of the coating.
3. We conducted a 1:1 dynamic simulation test on a C/C composite brake disc coated with gradient coating. The brake disc exhibited a good appearance after the test, without oxidation or damage. This shows that the gradient coating can meet the requirements for aircraft use. The preparation process of the coating is simple and low-cost and can meet the demands of mass production.

## Figures and Tables

**Table 1:** Raw materials and their main indexes.

	Category	Key Technical Indicators		Suppliers
		Granularity/Purity	Mass Fraction	
Layer I	B_4_C	(8 to 10) μm	10–15%	Mudanjiang Boron Carbide Abrasives Co., Ltd. (Mudanjiang City, China)
	Si (OC2H5)4	Analytically pure	30–35%	Sinopharm Chemical Reagent Co., Ltd. (Shanghai, China)
Layer II	SiO_2_	(0.15 to 0.25) μm	5–10%	Shanghai TOPKEN Building Material Co., Ltd. (Shanghai, China)
	B_2_O_3_	98% or higher	10–15%	Sinopharm Chemical Reagent Co., Ltd.
	Na_2_B_4_O_7_.10H_2_O	99.5% or higher	10–15%	Sinopharm Chemical Reagent Co., Ltd.

**Table 2:** Test results for dynamic oxidation resistance of C/C sample with gradient coating from 900 °C to room temperature.

Sample No.	Weight Loss Ratio	Sample No.	Weight Loss Ratio
(1)	−0.11%	(7)	−0.19%
(2)	−0.31%	(8)	−0.35%
(3)	−0.06%	(9)	−0.48%
(4)	−0.41%	(10)	−0.49%
(5)	−0.31%	(11)	−0.06%
(6)	−0.25%	(12)	−0.47%

## Data Availability

Data are contained within the article.
